# A novel recurrence-associated metabolic prognostic model for risk stratification and therapeutic response prediction in patients with stage I lung adenocarcinoma

**DOI:** 10.20892/j.issn.2095-3941.2020.0397

**Published:** 2021-08-15

**Authors:** Chengming Liu, Sihui Wang, Sufei Zheng, Xinfeng Wang, Jianbin Huang, Yuanyuan Lei, Shuangshuang Mao, Xiaoli Feng, Nan Sun, Jie He

**Affiliations:** 1 Department of Thoracic Surgery, National Cancer Center/National Clinical Research Center for Cancer/Cancer Hospital, Chinese Academy of Medical Sciences and Peking Union Medical College, Beijing 100021, China; 2 Department of Pathology, National Cancer Center/National Clinical Research Center for Cancer/Cancer Hospital, Chinese Academy of Medical Sciences and Peking Union Medical College, Beijing 100021, China

**Keywords:** Lung adenocarcinoma, stage I, recurrence, metabolic signature, immune landscape

## Abstract

**Objective:**

The proportion of patients with stage I lung adenocarcinoma (LUAD) has dramatically increased with the prevalence of low-dose computed tomography use for screening. Up to 30% of patients with stage I LUAD experience recurrence within 5 years after curative surgery. A robust risk stratification tool is urgently needed to identify patients who might benefit from adjuvant treatment.

**Methods:**

In this first investigation of the relationship between metabolic reprogramming and recurrence in stage I LUAD, we developed a recurrence-associated metabolic signature (RAMS). This RAMS was based on metabolism-associated genes to predict cancer relapse and overall prognoses of patients with stage I LUAD. The clinical significance and immune landscapes of the signature were comprehensively analyzed.

**Results:**

Based on a gene expression profile from the GSE31210 database, functional enrichment analysis revealed a significant difference in metabolic reprogramming that distinguished patients with stage I LUAD with relapse from those without relapse. We then identified a metabolic signature (i.e., RAMS) represented by 2 genes (*ACADM* and *RPS8*) significantly related to recurrence-free survival and overall survival times of patients with stage I LUAD using transcriptome data analysis of a training set. The training set was well validated in a test set. The discriminatory power of the 2 gene metabolic signature was further validated using protein values in an additional independent cohort. The results indicated a clear association between a high risk score and a very poor patient prognosis. Stratification analysis and multivariate Cox regression analysis showed that the RAMS was an independent prognostic factor. We also found that the risk score was positively correlated with inflammatory response, the antigen-presenting process, and the expression levels of many immunosuppressive checkpoint molecules (e.g., PD-L1, PD-L2, B7-H3, galectin-9, and FGL-1). These results suggested that high risk patients had immune response suppression. Further analysis revealed that anti-PD-1/PD-L1 immunotherapy did not have significant benefits for high risk patients. However, the patients could respond better to chemotherapy.

**Conclusions:**

This study is the first to highlight the relationship between metabolic reprogramming and recurrence in stage I LUAD, and is the first to also develop a clinically feasible signature. This signature may be a powerful prognostic tool and help further optimize the cancer therapy paradigm.

## Introduction

Lung cancer remains the most prevalent malignant tumor that seriously threatens human health worldwide, with approximately 2.1 million new cases and 1.8 million deaths each year^1^. Non-small cell lung cancer (NSCLC) is the predominant histological tumor type found in patients. Of the cases of NSCLC, approximately two-thirds are lung adenocarcinoma (LUAD)^2^. LUAD is diagnosed during the advanced or metastatic stages in most patients. However, the incidence of early-stage LUAD diagnosis is increasing sharply as low dose computed tomography (LDCT) screening has been more widely adopted in recent years^3–5^. Except for some pathology result-based high risk patients that require adjuvant chemotherapy, complete surgical resection is the recommended curative treatment for early stage LUAD (including stages I and II)^6,7^. However, for stage I LUAD, a clinical staging system seems to be only a weak predictor of relapse risk and long-term survival probability^8^. Large clinical trials have not found a significant benefit among unselected patients with stage I LUAD for whom the toxic effects associated with chemotherapy outweigh the potential survival benefits. Therefore, curative surgery remains the most popular therapeutic strategy to improve the prognoses of patients with stage I LUAD^5,9,10^. Nevertheless, high percentages of postoperative relapse or metastasis present a challenge for the long-term overall survival of patients with stage I LUAD^11^. Up to 30% experience tumor recurrence or metastasis within 5 years after curative surgery^12^. Therefore, robust discrimination criteria are needed that can be used to categorize stage I LUAD tumors after local resection and determine which patients have tumors at high risk for recurrence and could benefit from the use of adjuvant systemic therapy. Patients at low risk of tumor recurrence could be spared the use of this additional therapy.

Recently, there is increasing consensus that cancer metabolism is recognized as a well-established hallmark of tumor cells and also as an emerging source of novel potential drug targets^13,14^. Cancer cells must reprogram their metabolism to support various kinds of steps during carcinogenesis and cancer progression^15^. For example, they need to regulate metabolic programs to meet energy demands associated with rapid proliferation and migration. Cancer metabolism is modified partly by changes in cancer cell signaling and transcriptional programs activated by alterations in oncogenes or tumor suppressor genes^16^. Many commonly-mutated genes associated with LUAD regulate metabolic programs (e.g., *TP53, KRAS, EGFR, KEAP1*, and *PIK3CA, PTEN*)^17^. Specific driver mutations are associated with tumor progression, and KRAS mutations are related to disease recurrence in patients with stage I LUAD^18^. Thus, we examined whether metabolism-related genes could be a recurrence-associated gene signature that predicted recurrence in stage I LUAD.

In this study, we performed gene set enrichment analysis (GSEA) on gene expression profiles from the GSE31210 dataset. We found significant differences in metabolic reprogramming that could be used to distinguish stage I LUAD tumors with a relapse from those without a relapse. We then developed a recurrence-associated metabolic signature (RAMS) in 162 stage I LUAD samples from the GSE31210 dataset. This signature was validated using an independent set of 81 tumor samples from the GSE30219 dataset. To further test the reliability and practical application of the signature, we validated its prognostic power using protein values for selected genes and recurrence free survival (RFS) data for patients with stage I LUAD in a cohort recruited from the Cancer Hospital/Institute, Chinese Academy of Medical Sciences (CICAMS). Considering the promising prospects for immunotherapy in patients with lung cancer, we also comprehensively analyzed the clinical significance, immune checkpoint profiles, and immune cell infiltration of the novel RAMS. The results of this analysis provides the opportunity to further optimize the paradigm of cancer therapy, particularly of immunotherapy, and effectively reduce the relapse percentage of these tumors.

## Materials and methods

### Public gene expression datasets

We collected information on 243 cases of stage I LUAD cases with RFS data from 2 different Gene Expression Omnibus (GEO, http://www.ncbi.nlm.nih.gov/geo) datasets (162 cases from GSE31210 and 81 cases from GSE30219). The transcriptome data were log_2_ transformed and standardized across patients using a quantile normalized method. All corresponding characteristics of enrolled patients and clinical outcomes were publicly obtainable. The results for the patient information from the 2 cohorts are presented in **Supplementary Table S1**.

### Construction and validation of RAMS

We extracted 2,031 metabolism-related genes from the publicly accessible ccmGDB database (http://bioinfo.mc.vanderbilt.edu/ccmGDB)^19^. We then performed univariate Cox proportional hazards regression modeling to evaluate their prognostic value for RFS using stage I LUAD data from the GSE31210 dataset. Based on minimal criteria, a least absolute shrinkage and selection operator (LASSO) Cox proportional hazards regression model was used to select the genes with the largest predictive values. Next, we used a multivariate Cox proportional hazards regression model to determine the target genes that formed a RAMS for prognostication. We designed a formula to calculate the RAMS value of each patient, which included weighting the normalized expression value of the target genes by their respective coefficients. In the formula, the expression values of the target genes were normalized with a mean value = 0 and a standard deviation (SD) = 1 to obtain a uniform cut-off value to assign patients into low risk or high risk groups^20^. The prognostic power of the novel RAMS for RFS and overall survival (OS) was evaluated in three different cohorts using receiver operating characteristic (ROC) curve and Kaplan-Meier survival analyses. We also performed univariate and multivariate Cox regression analyses to determine whether RAMS was an independent prognostic risk factor.

### Patients in the CICAMS cohort and specimen collection

The CICAMS cohort enrolled 74 NSCLC patients who underwent radical surgery with systematic lymph node dissection from December 2012 to December 2013. The tumors of all eligible patients were diagnosed as stage I LUAD based on pathological characteristic (American Joint Committee on Cancer 8th TNM system). The patients did not undergo any preoperative treatments, such as chemotherapy or radiotherapy. The results for the clinicopathological information of the included patients are presented in **Supplementary Table S1**. Follow-up information was obtained through hospital visits or telephone contact with the patients or their relatives. The follow-up schedule consisted of a clinic visit every 3 months during the first 2 years, every 3–6 months from the third to fifth years, and at 1 year intervals thereafter. The Ethics Committee of CICAMS approved this study (approval number CH-L-043). All enrolled patients signed the written informed consent form before the study, in accordance with local ethics committee oversight.

### Immunohistochemistry (IHC) analysis

Paraffin-embedded LUAD samples from the CICAMS cohort were collected to examine the protein levels of 2 metabolic genes. Expression of ACADM and RPS8 were detected by IHC using an ACADM assay (anti-human ACADM rabbit recombinant monoclonal antibody, ab92461, Abcam, Cambridge, MA, USA) and a RPS8 assay (anti-human RPS8 rabbit polyclonal recombinant antibody, 18228-1-AP, Proteintech, Rosemont, IL, USA). All IHC slides were evaluated by 2 experienced pathologists, according to previously published evaluation criteria^20–22^. Each pathologist was blinded to the clinical parameters. The staining score of each sample was calculated using the following: staining score = staining intensity × percentage of positive tumor cells × 100. Staining intensity was scored according to the following: no color development was rated as 0 (negative), pale yellow as 1 (weak), yellow as 2 (moderate), and brown yellow as 3 (strong). Ten randomly-chosen fields were examined using a high power microscope (×400). The average value was used to calculate the percentage of tumor cells that positively stained, when compared with all tumor cells in the field of view. The results for representative staining images of ACADM and RPS8 at different levels are presented in **Supplementary Figure S1**.

### Immune cell infiltration analysis

The deconvolution algorithm, CIBERSORT (https://cibersort.stanford.edu/), was performed to calculate the fractions of 22 tumor-infiltrated immune cells in each sample, based on transcriptome data. The results were further filtered using *P* < 0.05^23,24^. The gene expression profile data was standardized across samples using a quantile-normalization method to remove effects of confounding variables.

### Clinical drug response prediction

Tumor Immune Dysfunction and Exclusion modeling (TIDE, http://tide.dfci.harvard.edu) was used to estimate TIDE prediction scores. Normalized mRNA data from each sample were included as model inputs. Patients whose TIDE prediction scores were greater than zero were identified as responders. Patients whose TIDE prediction scores were less than zero were identified as nonresponders. Transcriptome data were standardized across samples using a quantile-normalization method. The expression value of each gene was normalized by subtracting the average value among all samples. A zero value implied an average level of expression^25^.

After selecting frequently used clinical drugs from the Genomics of Drug Sensitivity in Cancer (GDSC) (https://www.cancerrxgene.org/) database, we performed the prediction analysis using the R package “pRRophetic” (https://github.com/paulgeeleher/pRRophetic). The analysis used ridge regression to estimate the half-maximal inhibitory concentration (IC_50_) of each sample. The prediction accuracy of the model was tested using 10-fold cross-validation based on a GDSC training set. All parameters in the program were set based on the default values, and duplicate gene expression was identified as the mean value^26,27^.

### Statistical analysis

Prism software (version 5.0; GraphPad, San Diego, CA, USA) and R software (version 3.6.0; The R Foundation, Vienna, Austria) were used to perform the statistical analyses. The survival curves for RFS and OS from the Kaplan-Meier survival analyses were compared using log-rank tests. Chi-square and Mann-Whitney U tests were used for between-group comparisons. All reported *P*-values were two-tailed. For all analyses, a value of *P* < 0.05 was considered a statistically significant result, unless otherwise specified.

## Results

### Relationships between relapse and metabolic phenotypes in stage I LUAD

**Figure 1:**
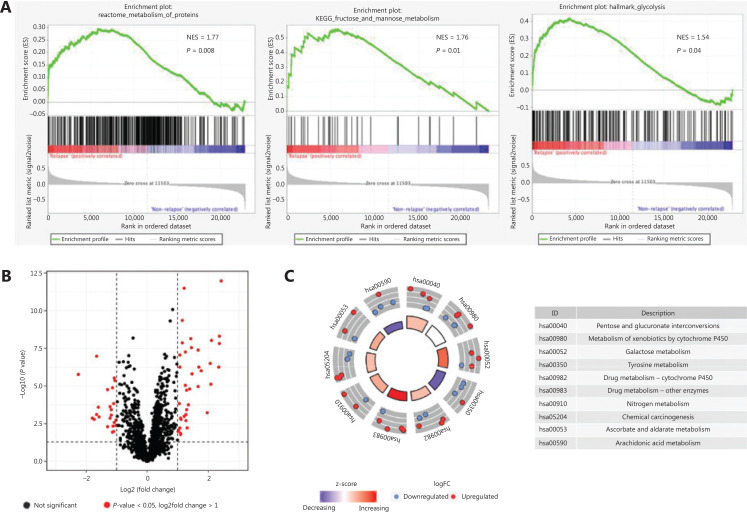
Relationships between relapse and metabolic phenotype in stage I LUAD. (A) Significant enrichment of metabolic pathways in relapsed patients with stage I LUAD, compared with non-relapsed patients. NES: normalized enrichment score. (B) Volcano plot of 64 metabolism-related genes differentially expressed in stage I LUAD samples with and without relapsed tumors. (C) Functional analysis of 64 metabolism-related genes.

To examine the distinct features of biological processes in relapsed stage I LUAD compared with non-relapsed tumors, we analyzed gene expression profiles in a cohort of 97 stage I LUAD tumors from GSE31210. We compared tumors from cases who remained recurrence-free for a minimum of 5 years with those from cases whose disease relapsed within 2 years of complete resection. GSEA of LUAD samples with (*n* = 17) and without (*n* = 80) relapsed tumors at baseline was performed. Except for hypoxia and immune responses, the GSEA results revealed that tumor relapse was strongly correlated with positive regulation of diverse metabolic pathways, including the metabolism of proteins pathway (NES = 1.77, *P* = 0.008), the fructose and mannose metabolism pathway (NES = 1.76, *P* = 0.01), and the glycolysis pathway (NES = 1.54, *P* = 0.04) (**Figure 1A**). To further investigate the associations between relapse and metabolic phenotype in stage I LUAD, we analyzed the expression profiles of 2,031 metabolism-related genes extracted from the ccmGDB database. Among these genes, 64 were found to be differentially expressed in LUAD samples with and without relapsed tumors (**Figure 1B**). Next, a Kyoto Encyclopedia of Genes and Genomes (KEGG) enrichment analysis was used to identify metabolic pathways associated with these 64 significant genes. The genes were involved in drug metabolism, galactose metabolism, and pentose and glucuronate interconversions (**Figure 1C**).

### Development of RAMS for stage I LUAD in the training cohort

**Figure 2:**
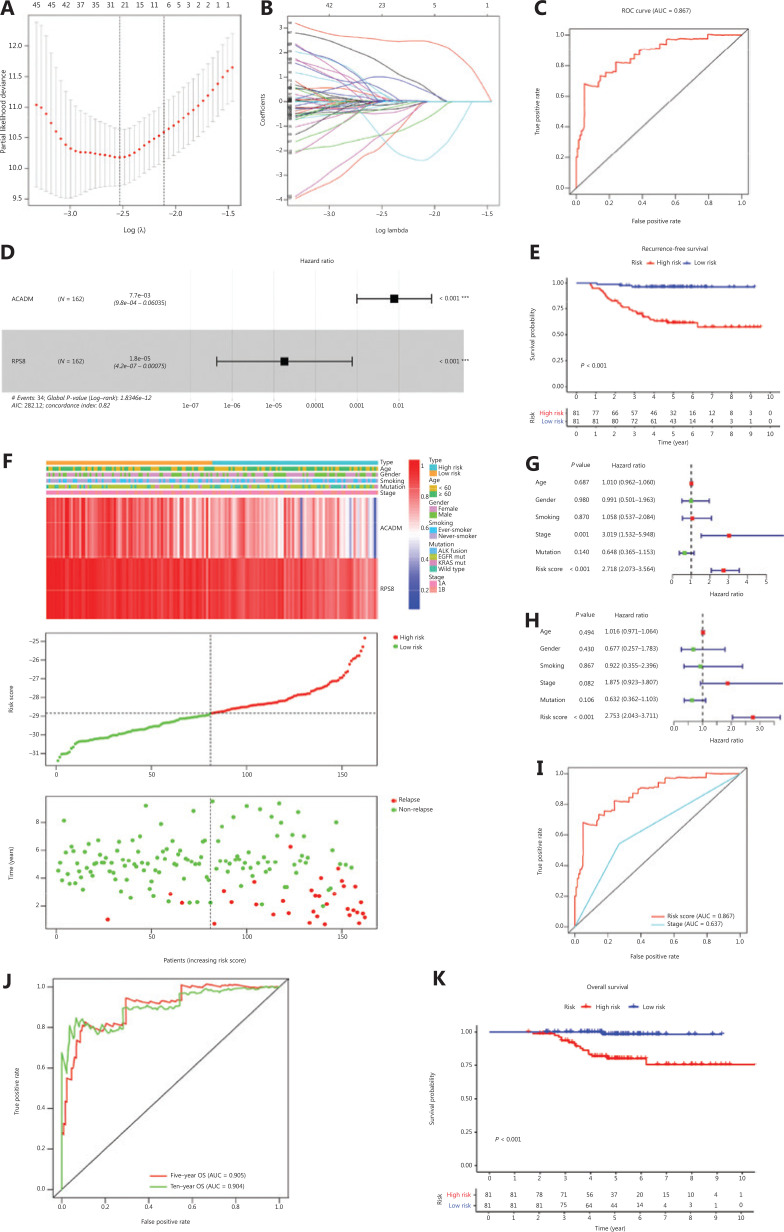
Development of RAMS for stage I LUAD in the training cohort. (A and B) The LASSO Cox proportional hazards regression model identified 23 genes most related to recurrence free survival (RFS). (C) Receiver operating characteristic (ROC) curve analysis of the RAMS for RFS. (D) Prognostic values of 2 selected genes using multivariate Cox proportional hazards regression analysis. (E) Kaplan-Meier survival curves of RFS for patients with stage I LUAD based on the RAMS. (F) Heat map of 2 gene expression profiles, risk score distributions, and recurrence status of each patient in the high and low risk groups. (G and H) Univariate (G) and multivariate (H) regression analyses of the associations between RAMS and clinical variables for the predictive value of RFS. (I) Performance was compared between RAMS and stages based on ROC curve analysis. (J) Time-dependent ROC curve analysis of the RAMS for overall survival (OS). (K) Kaplan-Meier survival curves of OS for patients with stage I LUAD, based on the RAMS. ***P < 0.001.

We found significant differences in metabolic reprogramming between patients with stage I LUAD with and without relapsed tumors at baseline. Therefore, we sought to develop a RAMS to improve prognostic prediction of stage I LUAD using a training cohort of 162 stage I LUAD samples from the GSE31210 database. Univariate Cox proportional hazards regression analysis was performed to identify metabolism-related genes correlated with RFS. Based on a value of *P* < 0.05, 727 genes out of 2,031 metabolism-related genes were identified as prognostic genes for RFS. We used LASSO Cox proportional hazards regression modeling to select genes with the greatest predictive values. Twenty-three genes were selected based on the minimum criteria (**Figure 2A and 2B**). Multivariate Cox regression analysis was then performed to further generate a RAMS for prognostication, and a novel prognostic signature consisting of only 2 genes (*ACADM* and *RPS8*) was built (**Figure 2D**). Subsequently, the risk score model for each patient was determined using the formula: risk score = −4.868 × normalized expression value of ACADM – 10.934 × normalized expression value of RPS8. Patients were assigned into low risk and high risk groups based on the optimal cut- off value (–28.842), which was obtained from the median value of the risk score model in the training cohort (**Figure 2F**).

To evaluate the predictive power of the novel RAMS, we calculated the area under the curve (AUC) values of the ROC, and performed Kaplan-Meier survival analysis. The results showed that the AUC value at 5-year RFS was 0.867 (**Figure 2C**). Patients in the high risk group had significantly worse RFS than those in the low risk group (*P* < 0.001; **Figure 2E**). To determine whether the risk score was an independent risk factor for RFS of patients with stage I LUAD, we performed univariate and multivariate Cox regression analyses of the training set. The Cox regression results indicated that both RAMS and stage were predictor factors (RAMS: *P* < 0.001; stage: *P* = 0.001; **Figure 2G**). However, the results after adjusting for clinicopathological factors including age, gender, smoking, stage, and mutation status indicated that RAMS was a significant independent prediction factor of RFS (*P* < 0.001; **Figure 2H**). The AUC value of RAMS was greater than for the stage (**Figure 2I**). A stratified analysis of stages IA and IB also revealed that a high risk score identified high risk patients (**Supplementary Figure S2**).

ROC and Kaplan-Meier survival analyses were also performed to test the robustness and practical application of RAMS for OS. The results indicated that the AUC values of the RAMS for OS were 0.905 at 5 years and 0.904 at 10 years (**Figure 2J**). High risk patients had an increased risk of mortality, compared with low risk patients (*P* < 0.001; **Figure 2K**). The results of the univariate and multivariate Cox regression analyses indicated that RAMS was an independent prognostic factor for OS (*P* < 0.001; **Supplementary Figure S5A–S5B**).

### Validation of RAMS for stage I LUAD in the test cohort

**Figure 3:**
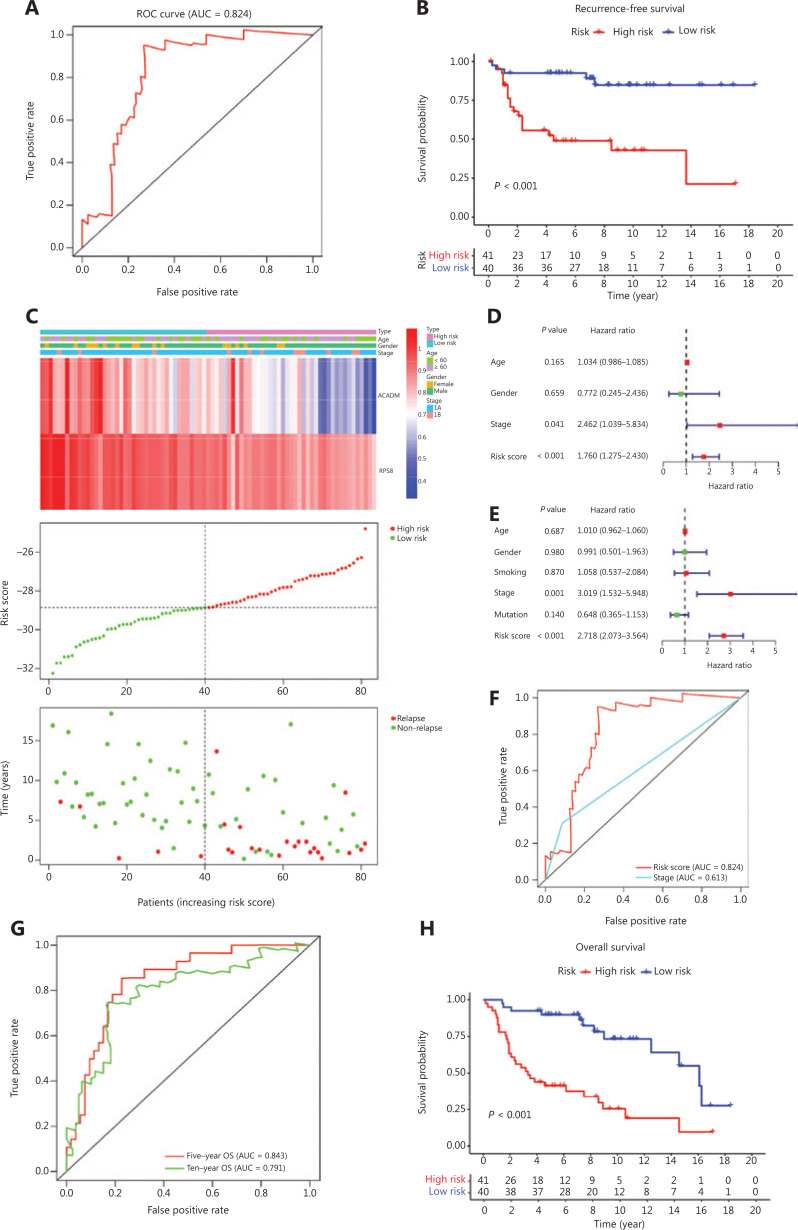
Validation of RAMS for stage I LUAD in the test cohort. (A) Receiver operating characteristic (ROC) curve analysis of the RAMS for recurrence free survival (RFS). (B) Kaplan-Meier survival curves of RFS for patients with stage I LUAD based on the RAMS. (C) Heat map of 2 gene expression profiles, risk score distributions, and recurrence status of each patient in the high and low risk groups. (D and E) Univariate (D) and multivariate (E) Cox regression analyses of the associations between RAMS and clinical variables for the predictive value of RFS. (F) The performance compared between RAMS and the stage was based on ROC curve analysis. (G) Time-dependent ROC curve analysis of the RAMS for overall survival (OS). (H) Kaplan-Meier survival curves of OS for patients with stage I LUAD based on the RAMS.

To verify the discriminatory power of RAMS for stage I LUAD, the same formula was used in the test set consisting of 81 cases from the GSE30219 database. Based on the cut-off values obtained from the training cohort, 81 patients were assigned to the low risk group (*n* = 40) or the high risk group (*n* = 41) (**Figure 3C**). The RAMS for stage I LUAD in the test cohort was identified as a robust prognostic model; its AUC value at a 5-year RFS was 0.824 (**Figure 3A**). Kaplan-Meier survival analysis showed that patients with a high risk score had significantly poorer RFS than those with a low risk score (*P* < 0.001) (**Figure 3B**). Due to a lack of stage IB cases, we only confirmed that a high risk score identified high risk patients using stage IA patients in the GSE31209 database (**Supplementary Figure S3**). Next, we examined whether RAMS was an independent risk factor of RFS for patients with stage I LUAD in the test set. We performed univariate and multivariate Cox regression analyses using the data from 81 patients. The results revealed that both RAMS and stage were prediction factors (RAMS: *P* < 0.001; stage: *P* = 0.041) (**Figure 3D**), and were also significant independent predictor factors of RFS (RAMS: *P* < 0.001; stage: *P* = 0.001) (**Figure 3E**). However, the AUC value of RAMS was greater than for the stage (**Figure 3F**). These results suggested that the RAMS had greater discriminatory power. We also performed ROC and Kaplan-Meier survival analyses to confirm the prognostic value of RAMS for OS. The results indicated that the performance of RAMS was stable; the AUC value was 0.843 at 5 years and 0.791 at 10 years (**Figure 3H**). The results of the Kaplan-Meier survival analysis suggested that patients in the high risk group had significantly poorer OS than those in the low risk group (*P* < 0.001) (**Figure 3G**). We also performed univariate and multivariate Cox regression analyses and found that RAMS was a significant independent prognostic factor of OS (*P* < 0.001; **Supplementary Figure S5C–S5D**).

### Validation of RAMS for stage I LUAD in the CICAMS cohort

**Figure 4:**
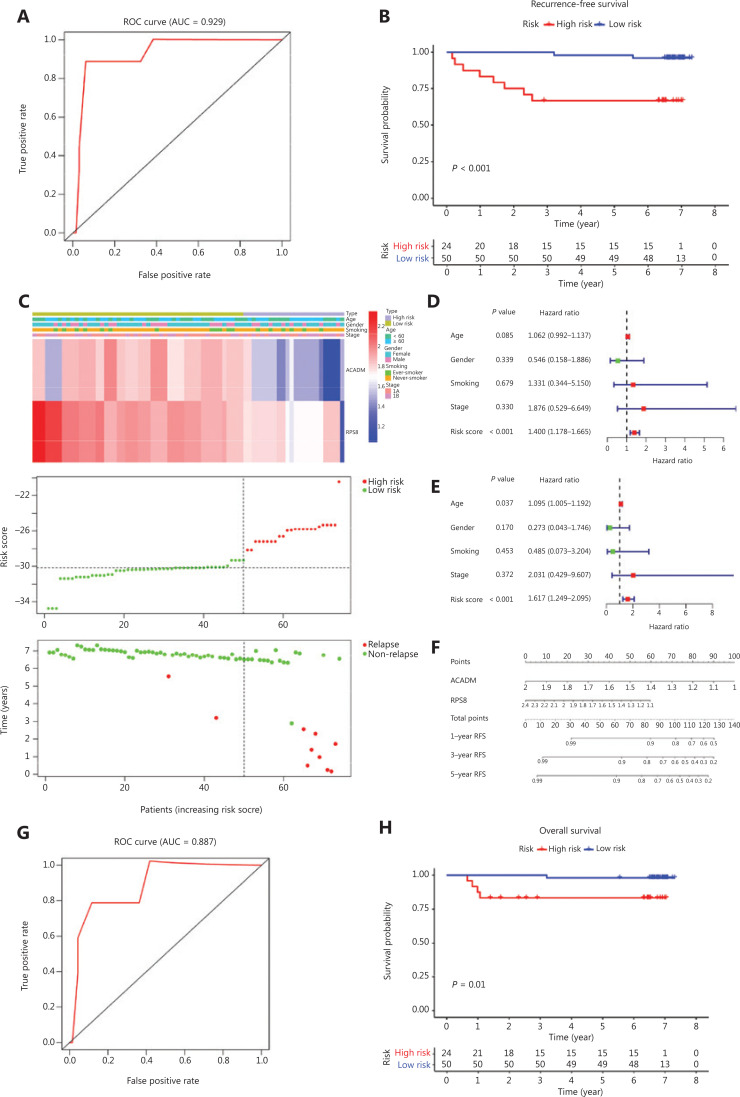
Validation of RAMS for stage I LUAD in the CICAMS cohort. (A) Receiver operating characteristic (ROC) curve analysis of the RAMS for recurrence free survival (RFS). (B) Kaplan-Meier survival curves of RFS for patients with stage I LUAD based on the RAMS. (C) Heat map of 2 gene expression profiles, risk score distributions, and recurrence status of each patient in the high and low risk groups. (D and E) Univariate (D) and multivariate (E) Cox regression analyses of the associations between the RAMS and clinical variables for the predictive value of RFS. (F) Nomogram to predict the 1-, 3-, and 5-year RFS of patients with stage I LUAD. (G) ROC curve analysis of the RAMS for overall survival (OS). (H) Kaplan-Meier survival curves of OS for patients with stage I LUAD based on the RAMS.

To further confirm the reliability and practical application of RAMS, we examined the predictive performance of RAMS using protein expression values in an independent cohort (CICAMS) of 74 patients with stage I LUAD. The protein expression levels of 2 metabolism-related genes (*ACADM* and *RPS8*) that formed the RAMS were detected using immunohistochemistry. The risk score of each patient was then calculated based on the same formula. As expected, the RAMS for stage I LUAD was still identified as a reliable prognostic model at the protein level; the AUC value for 5 years of RFS was 0.929 (**Figure 4A**). We then divided the 74 patients into a high risk group (*n* = 24) and a low risk group (*n* = 50) based on the same cut-off value (**Figure 4C**). Kaplan-Meier survival analysis showed a remarkable difference in RFS between the 2 groups (*P* < 0.001; **Figure 4B**). We also found that a high risk score identified high risk patients in a stratified analysis of stages IA and IB (**Supplementary Figure S4**). The results further confirmed that RAMS was an independent predictor factor of RFS (*P* < 0.001) (**Figure 4D and 4E**). Considering the simple and convenient application of RAMS, a predictive nomogram for RFS according to the normalized protein values of ACADM and RPS8 was also generated (**Figure 4F**). We also tested its ability to predict OS. We found that patients in the high risk group had shorter OS times than those in the low risk group (*P* = 0.01) (**Figure 4G**). We identified RAMS as a robust prognostic model; the AUC value was 0.887 for the 5-year OS. The results indicated that the prognostic ability of RAMS was independent of other risk factors for OS (*P* = 0.022) (**Supplementary Figure S5E–S5F**). We also constructed a predictive nomogram of OS according to the normalized protein values of ACADM and RPS8 (**Supplementary Figure S5G**).

### Association between RAMS and the immune response in stage I LUAD

**Figure 5:**
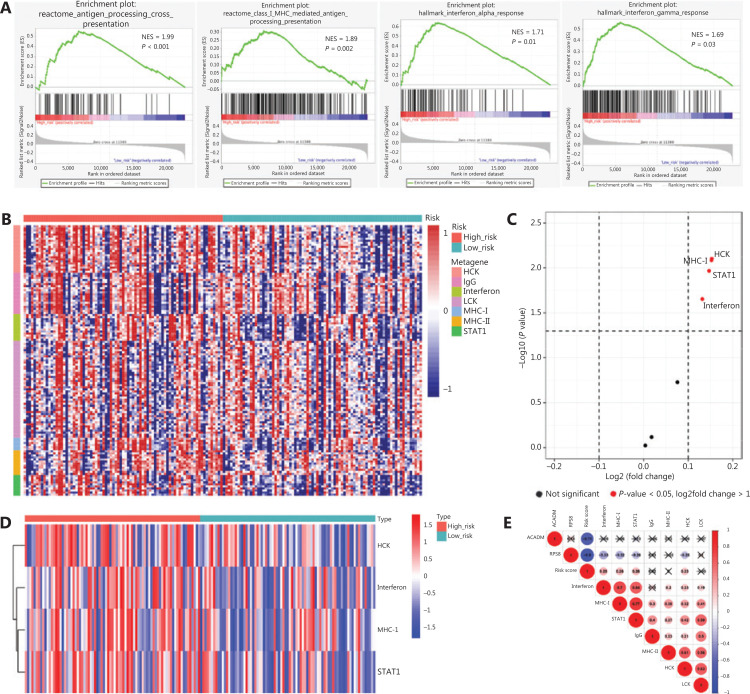
Associations between RAMS and immune response in stage I LUAD. (A) Significant enrichment of immune pathways between the high risk and low risk groups. NES: normalized enrichment score. (B) Heat map of the relationships between risk scores and 7 clusters of inflammatory metagenes. (C) Volcano plot of 4 clusters of inflammatory metagenes differentially enriched in the high and low risk groups. (D) Heat map of 4 clusters of inflammatory metagenes differentially enriched in the high and low risk groups. (E) Cross-correlogram based on Pearson’s correlation coefficient values between risk scores and 7 clusters of inflammatory metagenes.

To identify biological pathways related to RAMS, we divided 162 stage I LUAD cases from the GSE31210 database into a high risk group (*n* = 81) and a low risk group (*n* = 81) based on the cut-off values. We then performed a GSEA to determine the distinct features of biological processes between the 2 groups. The results indicated that patients at high risk had characteristics that were strongly associated with positive regulation of diverse immune pathways, including the antigen processing cross presentation pathway (NES = 1.99, *P* < 0.001), the class I MHC-mediated antigen processing presentation pathway (NES = 1.89, *P* = 0.002), the interferon alpha response pathway (NES = 1.71, *P* = 0.01), and the interferon gamma response pathway (NES = 1.69, *P* = 0.03) (**Figure 5A**).

To better understand the association between RAMS and immune response, we analyzed the expression of 7 previously described clusters of inflammatory metagenes (*HCK, IgG*, interferon*, LCK, MHC-I, MHC-II*, and *STAT1*) between the high risk and low risk groups (**Figure 5B**)^28^. Gene set variation analysis was performed to further characterize the expression of metagene clusters between the 2 groups. The analysis revealed a strong correlation between risk score and the hemopoietic cell kinase pathway, interferon response pathway, MHC class I processing pathway, and STAT1 signal transduction pathway (**Figure 5C**)^29^. The results for expression levels of 4 significantly differentially expressed metagene clusters for all samples between the 2 groups are presented in **Figure 5D**. To validate the findings and improve interpretation of the associations, we used a cross-correlogram to display correlations among these variables. The results showed that risk score had positive associations with HCK, interferon, MHC-I, and STAT1. However, RPS8 had a negative association with these 4 metagene clusters (**Figure 5E**).

### Correlation between RAMS and immune cell infiltration or immune checkpoint profiles in stage I LUAD

**Figure 6:**
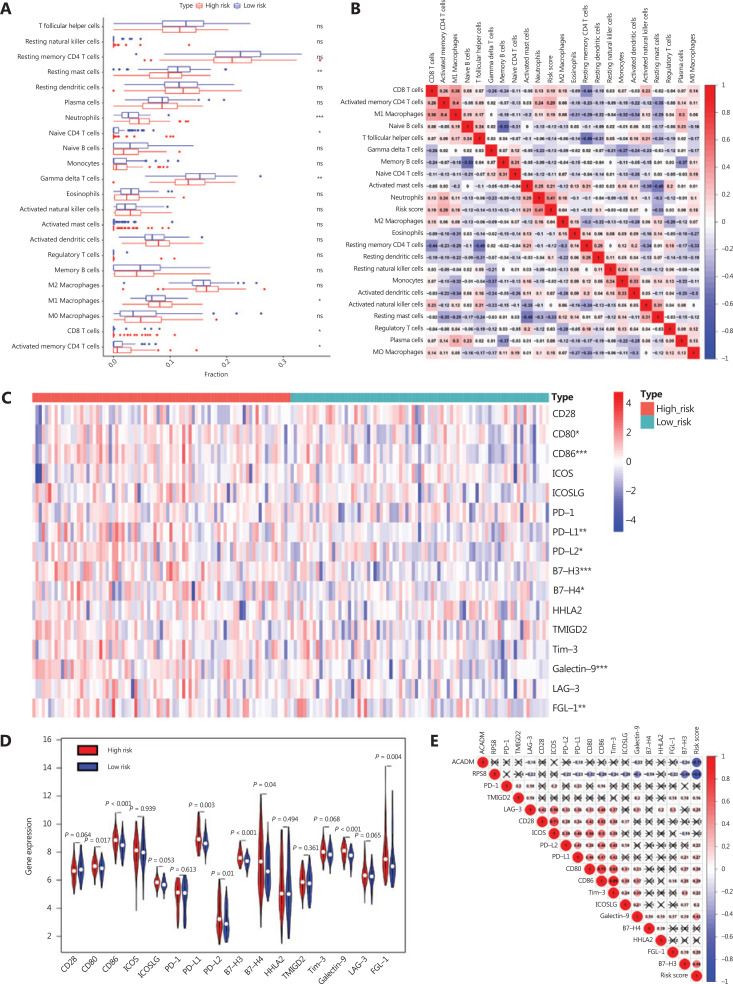
Correlation between RAMS and immune cell infiltration or immune checkpoint profiles in stage I LUAD. (A) Differences in immune cell infiltration abundances between the high risk and low risk groups. (B) Cross-correlogram based on Pearson’s correlation coefficient values between risk scores and 22 tumor-infiltrated immune cells. (C) Heat map of immune checkpoint profiles in the high and low risk groups. (D) Differences in expressions of immune checkpoint molecules between high risk and low risk groups. (E) Cross-correlogram was based on Pearson’s correlation coefficient values between risk scores and expressions of immune checkpoint molecules. *P < 0.05; **P < 0.01; ***P < 0.001.

Given that immune response is closely related to the immune cell landscape, we used a CIBERSORT algorithm to analyze intratumoral immune cell compositions between the high risk and low risk groups in the GSE31210 database. The results indicated that samples from high risk patients were characterized by neutrophil enrichment (*P* < 0.001) and a lack of gamma delta T cells and resting mast cells (*P* < 0.01) (**Figure 6A**). To further verify the association between risk score and immune cell infiltration, cross-correlograms were used to identify associations among these variables. The results consistently indicated that risk score was positively correlated with neutrophil infiltration level (*r* = 0.41) but was negatively correlated with resting mast cell level (*r* = –0.33) (**Figure 6B**).

Next, to gain new insights into the correlations between RAMS and immune checkpoint profiles, we included 16 immune checkpoint molecules in the analysis. We included the B7-CD28 family (CD28, CD80, CD86, ICOS, ICOSLG, PD-1, PD-L1, PD-L2, B7-H3, B7-H4, HHLA2, and TMIGD2) and several other hotspot immune checkpoint members (Tim-3, galectin-9, LAG-3, and FGL-1) (**Figure 6C**)^30–33^. The results indicated that higher levels of CD80, CD86, PD-L1, PD-L2, B7-H3, galectin-9, and FGL-1 were expressed in the high risk group (**Figure 6C and 6D**). We used a cross-correlogram to better characterize the associations between risk scores and the types of immune checkpoint molecules. We found that risk score had a positive correlation with B7-H3 (*r* = 0.49) and galectin-9 (*r* = 0.42) (**Figure 6E**).

### Relationship between RAMS and the drug response in stage I LUAD

**Figure 7:**
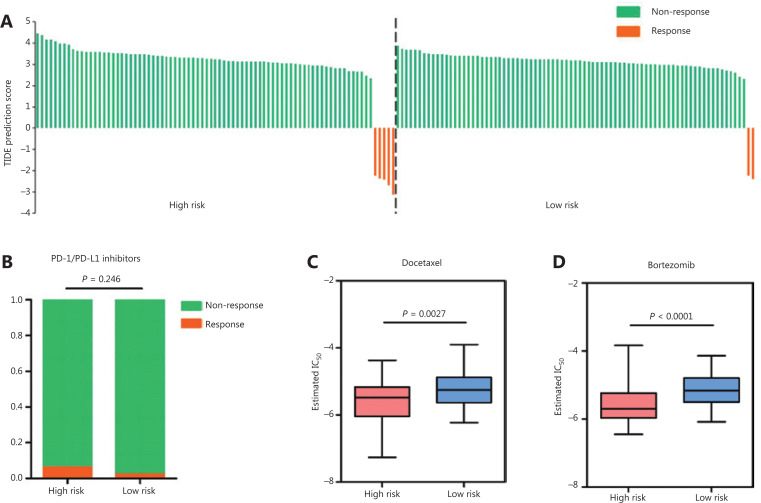
Relationships between RAMS and drug response in stage I LUAD. (A) Waterfall plot of TIDE prediction scores in the high and low risk groups. Red indicates a tumor that responded to therapy. Green indicates nonresponders. (B) A box plot evaluating immunotherapeutic responses between high risk and low risk patients using TIDE prediction scores. (C and D) Box plots evaluating responses to the chemotherapeutics docetaxel (C) and bortezomib (D) between high risk and low risk patients.

Because RAMS was highly related to immune response, immune checkpoint profiles, and immune cell infiltration, we investigated whether RAMS predicted the immunotherapeutic response of immune checkpoint inhibitors. The TIDE algorithm was used to predict immune checkpoint inhibitor response using pretreatment RNA-seq data between the high risk and low risk groups in the GSE31210 database. The objective response rates were 5 of 81 (6.17%) for the high risk and 2 of 81 (2.47%) for the low risk groups (**Figure 7A**). The results indicated that although high risk patients may respond better to anti-PD-1/PD-L1 immunotherapy, the difference was not statistically significant (*P* = 0.247) (**Figure 7B**).

Given that chemotherapy and target therapy are commonly used in the comprehensive treatment program for NSCLC, we tried to evaluate the sensitivity of many anti-cancer clinical drugs based on tumor gene expression levels using the R package “pRRophetic.” After estimating the IC_50_ for each sample in the GSE31210 cohort, we selected 2 drugs (docetaxel and bortezomib), which had significant response sensitivities for high risk cases, when compared with low risk cases (docetaxel: *P* = 0.0027; bortezomib: *P* < 0.0001; **Figure 7C and 7D**).

## Discussion

The percentage of patients with stage I LUAD has dramatically increased as the prevalence of LDCT use for screening has increased^34^. Surgery alone remains the standard treatment for these patients, but many are at substantial risk of relapse and death without adjuvant treatment^35^. Clinical staging, which is currently the best confirmed predictor of survival and a guide for treatment, seems to be only a weak predictor of relapse risk and long-term survival of patients with stage I LUAD^8^. A robust discrimination tool is therefore urgently needed to identify patients with stage I LUAD who could benefit from adjuvant systemic therapy.

Only a few studies have focused on the distinct features of biological processes in relapsed patients with stage I LUAD, when compared with non-relapsed patients. We therefore performed GSEA on gene expression profiles from the GSE31210 database to identify significant differences in metabolic reprogramming that could be used to distinguish patients with stage I LUAD tumors with relapses from those without relapses. Consistent with this finding, other studies have reported that cancer metabolism has an important role in the development of NSCLC, and also in the prognosis of NSCLC^17,36,37^. However, recurrence-associated prognostic models based on metabolism-related genes are lacking. In this study, we identified a novel metabolic signature (RAMS) significantly related to the RFS and OS of patients with stage I LUAD. We used transcriptome data analysis of a training cohort from the GSE31210 database, which was also well validated in a test cohort from the GSE30219 database. The subsequent IHC analysis of data from the additional independent CICAMS cohort further validated the protein level discriminatory power of RAMS.

In this study, 2 metabolism-related genes (*ACADM* and *RPS8*) were identified and included in the recurrence-associated prognostic model. They were negatively associated with favorable outcomes and were found to participate in tumor progression. ACADM (medium-chain acyl-CoA dehydrogenase) participates in the initial steps of the mitochondrial fatty acid beta-oxidation pathway and is involved in pathways associated with fatty acid metabolic disorders. These pathways are components of tumor transformation^38,39^. Low expression of ACADM is related to unfavorable outcomes and is likely involved in the tumorigenesis, invasion, and relapse of clear cell renal cell carcinoma; this finding is, to some extent, consistent with our results^39^. Another study also reported that attenuating ACADM activity accelerated cancer progression^40^. RPS8 (40S ribosomal protein S8; component of the ribosomal 40S subunit) participates in tumor development as a rate-limiting factor during translational control^41,42^. Translational regulation appears to have important roles in tumorigenesis, differentiation, and apoptosis, but abnormal translation also often induces malignant transformation in many cancers^43,44^. Although the molecular mechanism of low RPS8 expression in malignant tumors remains unclear, RPS8 and CDK11p46, which mainly co-localize in the nucleoplasm where pre-ribosomal subunits are formed, synergistically inhibit protein synthesis during the translation process and sensitize cells to FasL-induced apoptosis^41^. Our study is the first to show that low expression of ACADM and RPS8 was correlated with an unfavorable prognosis in patients with stage I LUAD. However, the elucidation of functional signaling processes of the 2 genes in LUAD needs further research.

Stratification analysis and multivariate Cox analysis found that RMAS was an independent prognostic factor for RFS in patients with stage I LUAD. Nevertheless, due to a lack of stage IB cases in the GSE30219 cohort, we only found that a high risk score identified high risk patients in the stratified analysis of stages IA and IB in the GSE31210 and CICAMS cohorts. We also compared the robustness of RAMS with clinical staging and found that the AUC value of RAMS was higher than the stage in 3 different cohorts. This result increased our confidence that in the future, RAMS will be an effective prognostic tool. Because postoperative relapse is the main reason for cancer-associated death in patients with stage I LUAD, we also investigated the predictive power of RAMS for long-term survival. As expected, we found that RAMS was significantly correlated with OS and was an independent prognostic factor in patients with stage I LUAD. However, because the data from the 3 cohorts were retrospective data, more prospective studies are needed to confirm the results and our conclusions.

To investigate the potential underlying mechanism of RAMS that discriminated high risk and low risk patients, we performed GSEA to determine the distinct features of the biological processes between the 2 groups. We found that high risk was strongly associated with positive regulation of diverse immune pathways. This result indicated that immune heterogeneity between the 2 groups may be the main cause of the difference in overall prognosis and cancer recurrence. A previous study also found that the immune response was largely shaped by cell metabolism^45^. We therefore examined RAMS-associated immune variations between the 2 groups. Analyses of 7 clusters of inflammatory metagenes, immune cell infiltration, and immune checkpoint profiles were used to provide additional insight into the immune landscapes associated with these 2 groups. We found that risk score had a positive relationship with inflammatory response (HCK, STAT1, and interferon), antigen-presenting process (MHC-I), and the expression levels of many immunosuppressive checkpoint molecules, including PD-L1, PD-L2, galectin-9, B7-H3, and FGL-1. These results suggested that high risk patients were in an immunosuppressive state. The immune systems of low risk patients involved high infiltration of gamma delta T cells, which indicated the presence of a relatively active anti-tumor immune response state.

Considering the pre-existing tumor immunity features of high risk patients, we further examined whether anti-PD-1/PD-L1 immunotherapy resulted in a survival benefit for these patients. Unfortunately, the results of TIDE algorithm prediction indicated that anti-PD-1/PD-L1 immunotherapy did not yield significant benefits for patients in the high risk group. There were 2 reasons for this result. First, tumors from high risk patients did not only express high levels of PD-L1and PD-L2. They also expressed high levels of other immunosuppressive checkpoint molecules, such as B7-H3, galectin-9, and FGL-1. Second, patients treated with immunotherapy were not included in this study. Therefore, the predictive ability of RAMS for immunotherapy response was evaluated indirectly. Further studies are needed to confirm these findings. It is important to note that using the GDSC database, we found that tumors from high risk patients could be more sensitive to commonly used chemotherapy (e.g., docetaxel). Therefore, high risk patients could receive chemotherapy after curative surgery to obtain a longer survival. Nevertheless, further comprehensive prospective studies are still needed.

## Conclusions

Overall, this study was the first to highlight the relationship between metabolic reprogramming and recurrence in stage I LUAD. We developed a novel RAMS model, which could serve as a powerful prognostic tool and potentially be used to guide the clinical management of patients with stage I LUAD.
