# Supplementary material for: A novel recurrence-associated metabolic prognostic model for risk stratification and therapeutic response prediction in patients with stage I lung adenocarcinoma
Source: Cancer Biol Med. 2021 Aug 15;18(3):734–49. doi: 10.20892/j.issn.2095-3941.2020.0397 (PMC8330534; doi:10.20892/j.issn.2095-3941.2020.0397)
Supplement: Supplementary file 1 [file cbm-18-734-s001.pdf]

Supplementary materials

Table S1 Demographic characteristics of stage I LUAD patients in 3 cohorts

Characteristics	GSE31210	GSE30219	CICAMS
Case No.	162	81	74
Gender, *N* (%)			
Male	69 (42.6)	17 (62.7)	26 (35.1)
Female	93 (57.4)	64 (37.3)	48 (64.9)
Age, *N* (%)			
< 60	70 (43.2)	37 (49.2)	34 (45.9)
≥ 60	92 (56.8)	44 (50.8)	40 (54.1)
Smoking Status, *N* (%)			
Never	90 (55.6)	–	56 (75.7)
Ever	72 (44.4)	–	18 (24.3)
Stage, *N* (%)			
IA	109 (67.3)	69 (67.8)	40 (54.1)
IB	53 (32.7)	12 (32.2)	34 (45.9)
Mutation, *N* (%)			
EGFR mutation	100 (61.7)	–	–
KRAS mutation	14 (8.6)	–	–
ALK fusion	2 (1.2)	–	–
Wild type	46 (28.5)	–	–

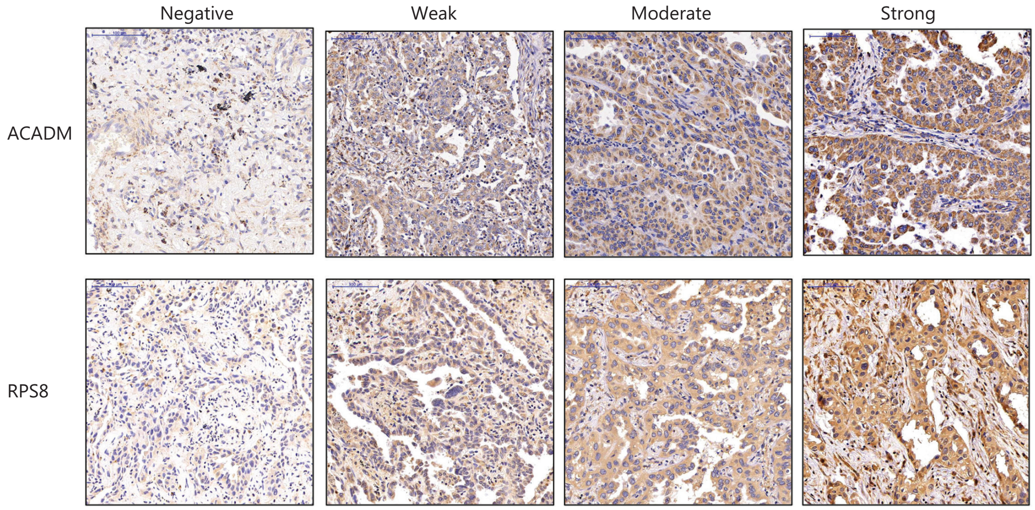

Figure S1 Representative staining images of ACADM and RPS8 at different levels.

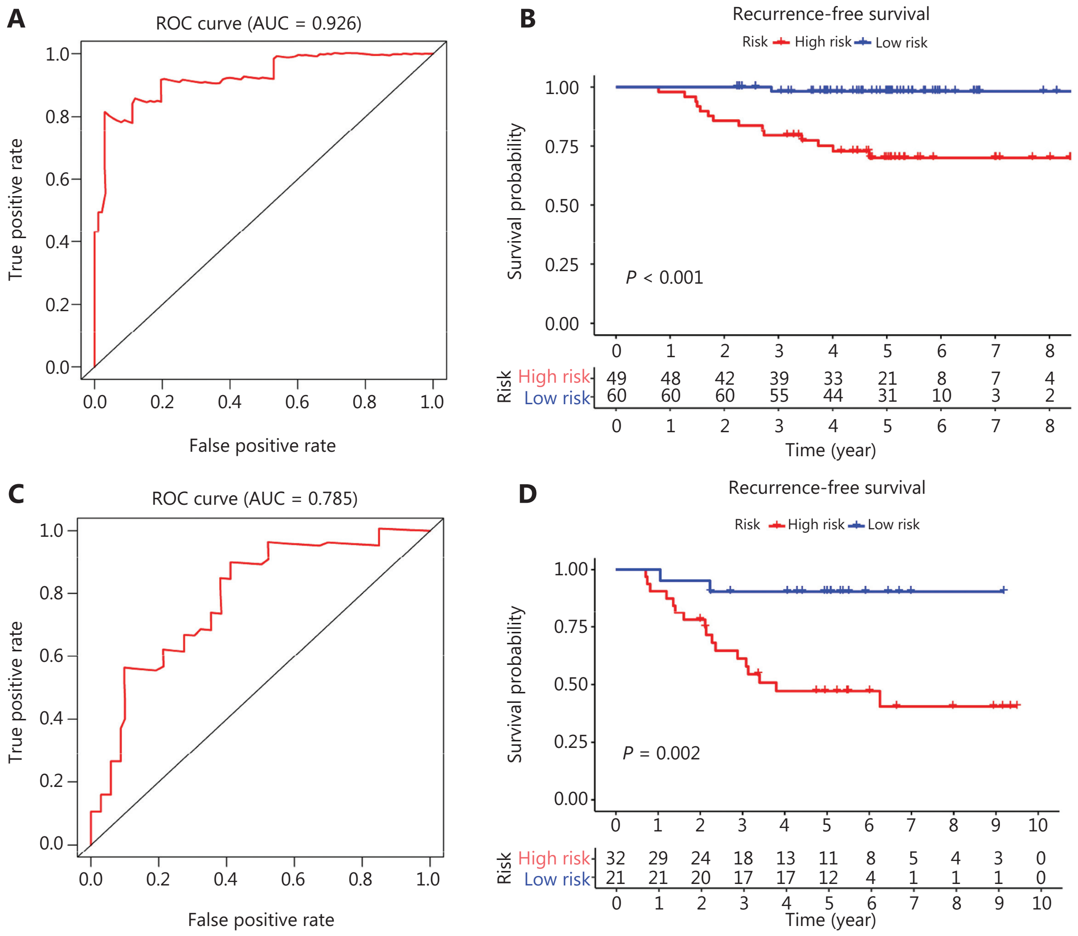

Figure S2 Stratification analysis of RAMS for its prognostic value in the GSE31210 set. (A) Receiver operating characteristic (ROC) curve analysis of the RAMS for recurrence free survival (RFS) in stage IA LUAD patients. (B) Kaplan-Meier survival curve of RFS for patients with stage IA LUAD based on the RAMS. (C) ROC curve analysis of the RAMS for RFS in stage IB LUAD patients. (D) Kaplan-Meier survival curve of RFS for patients with stage IB LUAD based on the RAMS.

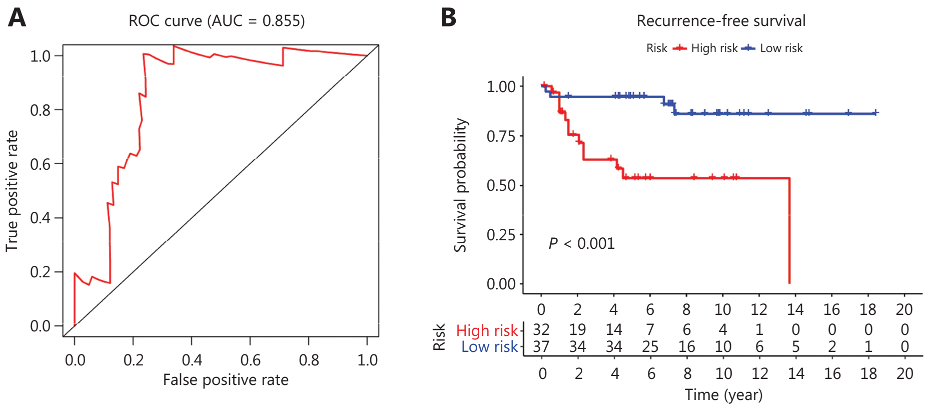

Figure S3 Stratification analysis of RAMS for its prognostic value in the GSE30219 set. (A) Receiver operating characteristic curve analysis of the RAMS for recurrence free survival (RFS) in stage IA LUAD patients. (B) Kaplan-Meier survival curve of RFS for patients with stage IA LUAD based on the RAMS.

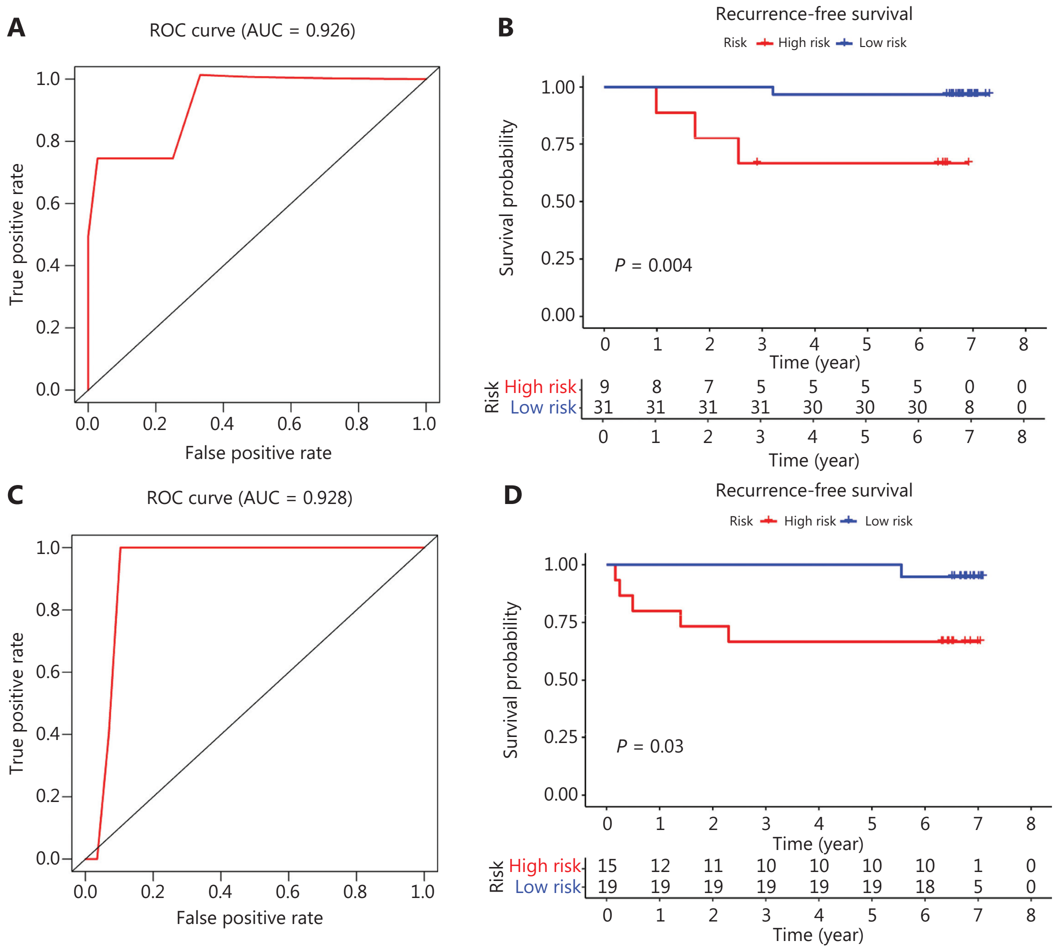

Figure S4 Stratification analysis of RAMS for its prognostic value in the CICAMS cohort. (A) Receiver operating characteristic (ROC) curve analysis of the RAMS for recurrence free survival (RFS) in stage IA LUAD patients. (B) Kaplan-Meier survival curve of RFS for patients with stage IA LUAD based on the RAMS. (C) ROC curve analysis of the RAMS for RFS in stage IB LUAD patients. (D) Kaplan-Meier survival curve of RFS for patients with stage IB LUAD based on the RAMS.

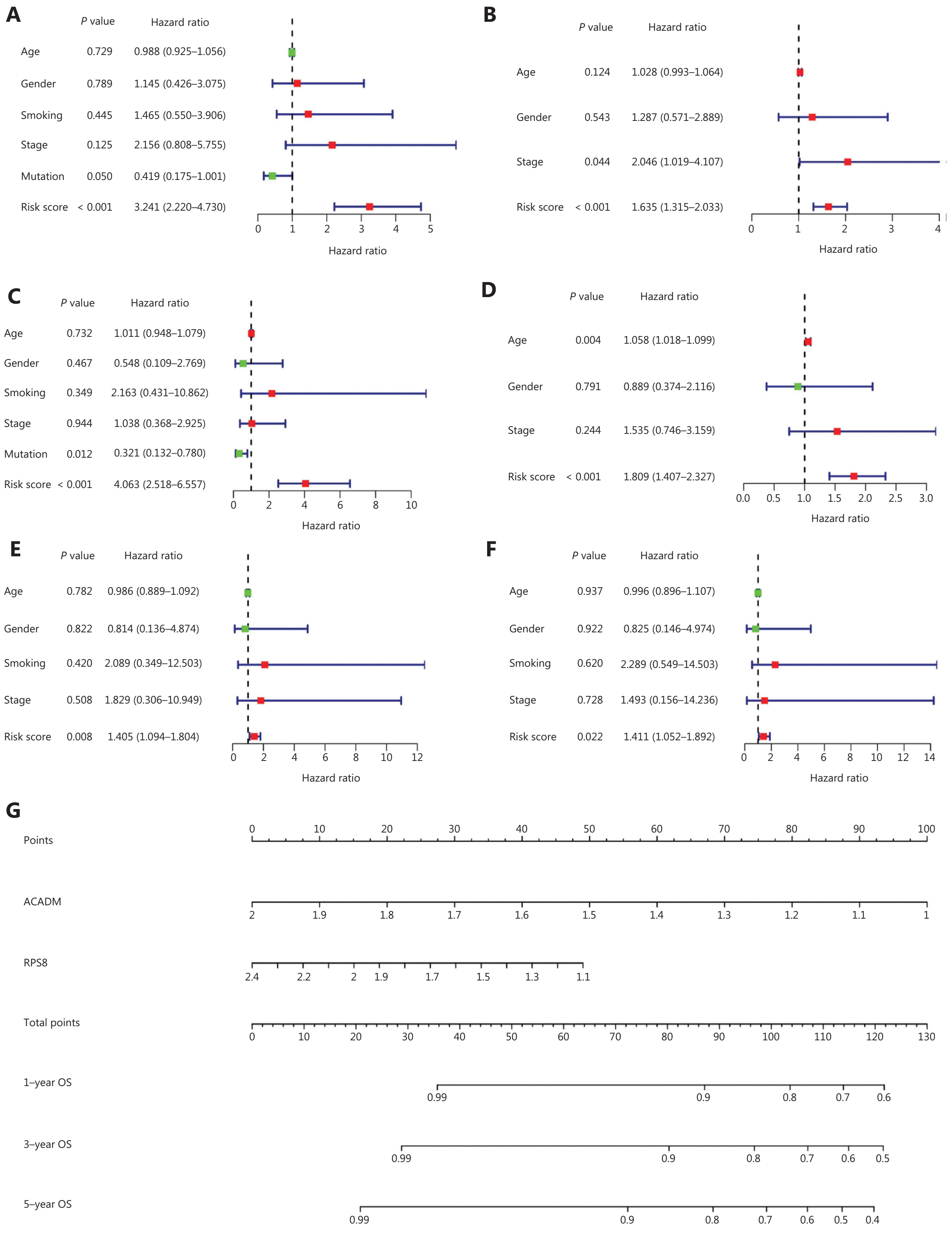

Figure S5 Univariable and multivariable Cox regression analyses of RAMS and clinical characteristics with overall survival (OS) in 3 cohorts. (A and B) Univariate (A) and multivariate (B) Cox regression analyses of the association between the RAMS and clinical variables regarding predictive value of OS in the GSE31210 set. (C and D) Univariate (C) and multivariate (D) Cox regression analyses of the associations between RAMS and clinical variables for predictive values of OS in the GSE30219 set. (E and F) Univariate (E) and multivariate (F) Cox regression analyses of the associations between RAMS and clinical variables for predictive values of OS in the CICAMS cohort. (G) Nomogram to predict the 1-, 3-, and 5-year OS of patients with stage I LUAD in the CICAMS cohort.
